# Supplementary material for: A tool to pulse-label yeast nuclear pore complexes in imaging and biochemical experiments
Source: eLife. 2026 Aug 10;14:RP108399. doi: 10.7554/eLife.108399 (PMC13456684; doi:10.7554/eLife.108399)
Supplement: Peer review history

# Editor report: eLife Assessment

**DOI:** 10.7554/eLife.108399.3.sa0

**Authors:** Elçin Ünal

**Affiliations:** University of California, Berkeley United States

**Keywords:** Convincing, Important

This **important** study introduces a non-perturbative pulse-labeling strategy for yeast nuclear pore complexes (NPCs), employing a nanobody-based approach in order to selectively capture Nup84-containing complexes for imaging and biochemical analysis. The data **convincingly** demonstrate that a short induction period (20 minutes to 1 hour) yields a strong and sustained signal, enabling affinity purification that faithfully recapitulates the endogenous Nup84 interactome. This tool offers a powerful framework for investigating NPC dynamics and associated interactomes through both imaging and biochemical assays.

# Referee report: Reviewer #1 (Public review)

**DOI:** 10.7554/eLife.108399.3.sa1

Summary:

The authors present a nanobody-based pulse-labeling system to track yeast NPCs. Transient expression of a nanobody targeting Nup84 (fused to NeonGreen or an affinity tag) permits selective visualization and biochemical capture of NPCs. Short induction effectively labels NPCs, and the resulting purifications match those from conventional Nup84 tagging. Crucially, when induction is repressed, dilution of the labeled pool through successive cell cycles allows the visualization of "old" NPCs (and potentially individual NPCs) providing a powerful view of NPC lifespan and turnover without permanently modifying a core scaffold protein.

Strengths:

(1) A brief expression pulse labels NPCs, and subsequent repression allows dilution-based tracking of older (and possibly single) NPCs over multiple cell cycles.

(2) The affinity-purified complexes closely match known Nup84-associated proteins, indicating specificity and supporting utility for proteomics.

Weakness:

Reliance on GAL induction introduces metabolic shifts (raffinose → galactose → glucose) that could subtly alter cell physiology or the kinetics of NPC assembly. As acknowledged by the authors, alternative induction systems (e.g., β-estradiol-responsive GAL4-ER-VP16) could be implemented as a way to avoid carbon-source changes.

Comments on revised version.

The authors have thoughtfully addressed all of my concerns. In particular, they have updated the proteomic analysis in Figure 1I, showing that they recover most NPC components (including basket Nups), including non-NPC proteins as controls, and providing all data as a supplementary table. These changes strengthen the authors conclusion and improve transparency. I have no further recommendations and congratulate the authors for their exciting work.

# Referee report: Reviewer #2 (Public review)

**DOI:** 10.7554/eLife.108399.3.sa2

Summary:

This preprint describes a practical and useful approach for labeling and tracking NPCs in situ, using a fluorescently conjugated nanobody that binds directly to the core scaffold nucleoporin Nup84 with nanomolar affinity. Useful applications including timelapse imaging, affinity purification, and proximity labeling are envisioned.

Strengths:

Clever use of a fluorescently conjugated nanobody that binds directly to the core scaffold nucleoporin Nup84 with nanomolar affinity.

# Referee report: Reviewer #3 (Public review)

**DOI:** 10.7554/eLife.108399.3.sa3

Summary:

Submitted to the Tools and Resources series, this study reports on the use of a single-domain antibody targeting the nucleoporin Nup84 to probe and track NPCs in budding yeast. The authors demonstrate their ability to rapidly label or pull down NPCs by inducing the expression of a tagged version of the nanobody (Fig. 1).

Strengths:

This tool's main strength is its versatility as an inexpensive, easy-to-set-up alternative to metabolic labelling or optical switching. This same rationale could, in principle, be applied to the study of other multiprotein complexes using similar strategies, provided that single-chain antibodies are available.

Weaknesses:

This approach has no inherent weaknesses, but it would be useful to verify in the future that this pulse labelling strategy can also be used to detect assembly intermediates, structural variants, or damaged NPCs, e.g. NPC clusters formed in some nucleoporin mutants.

Overall, the data clearly shows that Nup84 nanobodies are a valuable tool for imaging NPC dynamics and investigating their interactomes through affinity purification.

Comments on revised version.

None at this stage.

# Author comment: Author response

**DOI:** 10.7554/eLife.108399.3.sa4

**Authors:** Annemiek C Veldsink, Jonas S Fischer, Sophie Hell, Karsten Weis, Liesbeth M Veenhoff

**Affiliations:** University of Groningen Groningen Netherlands; ETH Zurich Zurich Switzerland; ETH Zurich Zurich Switzerland; ETH Zurich Zurich Switzerland; University of Groningen Groningen Netherlands

The following is the authors’ response to the original reviews.

**Public Reviews:**

**Reviewer #1 (Public review):**

Summary:

The authors present a nanobody-based pulse-labeling system to track yeast NPCs. Transient expression of a nanobody targeting Nup84 (fused to NeonGreen or an affinity tag) permits selective visualization and biochemical capture of NPCs. Short induction effectively labels NPCs, and the resulting purifications match those from conventional Nup84 tagging. Crucially, when induction is repressed, dilution of the labeled pool through successive cell cycles allows the visualization of "old" NPCs (and potentially individual NPCs), providing a powerful view of NPC lifespan and turnover without permanently modifying a core scaffold protein.

Strengths:

(1) A brief expression pulse labels NPCs, and subsequent repression allows dilution-based tracking of older (and possibly single) NPCs over multiple cell cycles.

(2) The affinity-purified complexes closely match known Nup84-associated proteins, indicating specificity and supporting utility for proteomics.

We thank the reviewer for this evaluation

Weaknesses:

(1) Reliance on GAL induction introduces metabolic shifts (raffinose -> galactose -> glucose) that could subtly alter cell physiology or the kinetics of NPC assembly. Alternative induction systems (e.g., β-estradiol-responsive GAL4-ER-VP16) could be discussed as a way to avoid carbon-source changes.

Indeed, this could be an improvement, and we mention the benefits of an inducible system that does not alter the cell’s metabolic state in the discussion on p.3.

(2) While proteomics is solid, a comprehensive supplementary table listing all identified proteins (with enrichment and statistics) would enhance transparency.

Indeed, we now provide source data showing LFQ intensities, fold-enrichment and statistics for all detected proteins.

(3) Importantly, the authors note that the method is particularly useful "in conditions where direct tagging of Nup84 interferes with its function, while sub-stoichiometric nanobody binding does not." After this sentence, it would be valuable to add concrete examples, such as experiments examining NPC integrity in aging or stress conditions where epitope tags can exacerbate phenotypes. These examples will help readers identify situations in which this approach offers clear advantages.

Indeed, we agree this would be useful. For example, in Nup1Δct and Nup60Δ mutants, GFP-tagging of Nup84 leads to slower growth and increased cell size (Ollivaud et al., BioRxiv). We have however not extensively tested nanobody expression in these mutants, and cannot conclude that it has no interfering effects. We therefore rephrased to “while sub-stoichiometric nanobody binding does may not, …”. Another situation where we find the nanobody-based labeling useful is when we want to assess the structural integrity (IPs) and localization (imaging) of NPCs in mutant strains, but prefer not to use tagged Nups in the actual experiments. In these cases, we transiently express the Nup84 nanobody to perform these checks, and then carry out the experiments without the nanobody to avoid any tag-related interference. We hence also added “,…or when the temporary introduction of a ZZ- or mNG-tagged nanobody allows assessment of the integrity or localization of mutant NPCs prior to performing experiments without the nanobody.

We thank the reviewer again for the constructive feedback and thoughts.

**Reviewer #2 (Public review):**

Summary:

This preprint describes a practical and useful approach for labeling and tracking NPCs in situ. While useful applications including timelapse imaging, affinity purification, or proximity labeling are envisioned, addressing some outstanding technical questions would give a clearer picture of the sensitivity and temporal resolution of this approach.

Strengths:

Clever use of a fluorescently conjugated nanobody that binds directly to the core scaffold nucleoporin Nup84 with nanomolar affinity.

We thank the reviewer for this evaluation

Weaknesses:

The decrease in nanobody labeling over 8 hours of chase period is interpreted to indicate that NPCs turn over during this time. However, it is also possible that the nanobody: Nup84 association is disrupted during mitosis by phosphorylation, other PTMs, or structural remodeling.

We thank the reviewer for this thought. It is actually not turnover that we propose to underly the decrease in nanobody labeling, but rather the dilution of labelled NPC to the daughter cell. The current data do not support the interpretation that the nanobody: Nup84 association is disrupted as proposed by the reviewer. The exchange of individual Nups, including Nup84, is slow with half-times in the order of hours (Hakhverdyan et al. 2021; Rabut, Doye, and Ellenberg 2004), and the nanobody: Nup84 association is very stable, namely in the nanomolar range (Nordeen et al. 2020). The association of nanobody with NPCs is thus expected to be very stable. Instead, dilution of labelled NPCs to the daughter - approximately 40% of the existing NPCs are transmitted to the daughter cell in each division (Zsok et al. 2024; Khmelinskii et al. 2010) – will lead to significant decreases in nanobody labelling over time. As the reviewer is likely aware, baker’s yeast NPCs – in contrast to mammalian NPCs - remain largely intact during cell division as there is no nuclear envelope breakdown.

We thank the reviewer again for the constructive feedback and thoughts.

**Reviewer #3 (Public review):**

Summary:

Submitted to the Tools and Resources series, this study reports on the use of a single-domain antibody targeting the nucleoporin Nup84 to probe and track NPCs in budding yeast. The authors demonstrate their ability to rapidly label or pull down NPCs by inducing the expression of a tagged version of the nanobody (Figure 1).

Strengths:

This tool's main strength is its versatility as an inexpensive, easy-to-set-up alternative to metabolic labelling or optical switching. This same rationale could, in principle, be applied to the study of other multiprotein complexes using similar strategies, provided that single-chain antibodies are available.

We thank the reviewer for this evaluation

Weaknesses:

This approach has no inherent weaknesses, but it would be useful for the authors to verify that their pulse labelling strategy can also be used to detect assembly intermediates, structural variants, or damaged NPCs.

We agree with the reviewer that it would be informative to see if VHH[Nup84] can bind its epitope in the context of an altered NPC structure but consider such studies to be beyond the scope of this study.

Overall, the data clearly show that Nup84 nanobodies are a valuable tool for imaging NPC dynamics and investigating their interactomes through affinity purification.

We thank the reviewer again for the constructive feedback and thoughts.

**Recommendations for the authors:**

**Reviewer #1 (Recommendations for the authors):**

(1) In Figure 1A, and although it is partially mentioned in the legend, it would be helpful to indicate precisely when cells are grown in raffinose, when galactose is added for induction, and when glucose is used to terminate expression.

We included “galactose” and “glucose” to Panel A to indicate induction and termination of expression, respectively.

(2) Related to the previous point, consider mentioning the GAL4-ER-VP16 (ADGEV) estradiol-inducible system as an optional strategy to avoid carbon shifts and potentially reduce cell-to-cell variability.

We mention the benefits of an inducible system that does not alter the cell’s metabolic state in the discussion on p.3

(3) Add a brief sentence explaining that the ZZ tag is derived from Protein A and binds IgG Fc.

This information is now added on p.2

(4) The statement "all Nups significantly coenriched with VHH[Nup84]-ZZ..." is likely inaccurate, since not all Nups are labeled in panels F-H, and some basket components are missing in panel I (particularly basket components such as Nup60, Nup1). Consider revising to "most Nups significantly coenriched...". In panel I, please include a clearly non-enriched protein as a visual reference for the color scale.

We are very grateful to the reviewer for pointing this out. We accidentally used a faulty filtering on the dataset to generate figure panel I, omitting several Nups that were reproducibly found in all replicas. All Nups, except for Gle1 and Pom33, were detected reproducibly.

We have made the following adjustments to the figure panel and accompanying text:

In Fig. 1I, we included the missing Nups and 5 proteins that co-purified with VHH[Nup84] but not specifically enriched, as the reviewer suggested. They cluster in a separate group and their abundance is not going up in time. We randomly selected these 5 proteins from the list of genes that were reproducibly found in all four timepoints.

For clarity, we removed the NTRs

We changed the text to “we found that all Nups, except Gle1 and Pom33, significantly coenriched with VHH[Nup84]-ZZ” on p.2.

We updated the methods section, describing the clustering method and how we selected the 5 random proteins

(5) Provide a supplementary spreadsheet with LFQ intensities, fold-enrichment, and statistics for all detected proteins. This will address questions about missing Nups and support transparency.

This information is now added as Source data Figure 1.

(6) Directly after the statement "Amongst others this is useful in conditions where direct tagging of Nup84 interferes with its function, while sub stoichiometric nanobody binding does not," it would be useful to include concrete instances, such as stress or aging conditions, where Nup84 tagging may sensitize NPC integrity.

Indeed, we agree this would be useful. For example, in Nup1Δct and Nup60Δ mutants, GFP-tagging of Nup84 leads to slower growth and increased cell size (Ollivaud et al., BioRxiv). We have however not extensively tested nanobody expression in these mutants and cannot conclude that it has no interfering effects. We therefore rephrased to “while sub-stoichiometric nanobody binding does may not, …”. Another situation where we find the nanobody-based labeling useful is when we want to assess the structural integrity (IPs) and localization (imaging) of NPCs in mutant strains, but prefer not to use tagged Nups in the actual experiments. In these cases, we transiently express the Nup84 nanobody to perform these checks and then carry out the experiments without the nanobody to avoid any tag-related interference. We hence also added “,…or when the temporary introduction of a ZZ- or mNG-tagged nanobody allows assessment of the integrity or localization of mutant NPCs prior to performing experiments without the nanobody.

(7) In panels K and L, since individual points correspond to biological replicates, overlaying a box plot obscures much of the data. Consider overlaying the means per replicate instead of box plots: see the "SuperPlots" approach for a clear explanation of how to present this (PMID: 32346721).

We thank the reviewer for the “SuperPlots” suggestion, and we agree that representing the data in this way improves the visualization of individual biological replicates. We have updated the summarizing overlay in figures in panel K and L to represent the means per replicate instead of boxplots.

(8) I spotted a few typos ("Lasty" ? "Lastly"; "in maintained" vs. "is maintained").

Thank you, these are corrected

Overall, this is a neat, well-executed methodological advance with clear value to the NPC field and potentially other complex assemblies. I look forward to seeing a revised version.

Thank you!

**Reviewer #2 (Recommendations for the authors):**

Based on the recent structural analyses and NPC modeling using this nanobody, how accessible is the Nup84 epitope expected to be within the fully assembled NPC? While the data shown indicate that nanobody labeling of NPCs is readily detectable, stating this clearly would help motivate the approach and interpret the resulting data.

We now included such a statement in the introduction on p.1.

The decrease of nanobody labeling over 8 hours of chase period is interpreted to indicate that NPCs turn over due to cell division during this time window. However, it is also possible that nanobody:Nup84 association is disrupted during mitosis by phosphorylation, other PTMs, or structural remodeling.

We thank the reviewer for this thought. It is actually not turnover that we propose to underly the decrease in nanobody labeling, but rather the dilution of labelled NPC to the daughter cell. The current data do not support the interpretation that the nanobody: Nup84 association is disrupted as proposed by the reviewer. The exchange of individual Nups, including Nup84, is slow with half-times in the order of hours (Hakhverdyan et al. 2021; Rabut, Doye, and Ellenberg 2004), and the nanobody: Nup84 association is very stable, namely in the nanomolar range (Nordeen et al. 2020). The association of nanobody with NPCs is thus expected to be very stable. Instead, dilution of labelled NPCs to the daughter - approximately 40% of the existing NPCs are transmitted to the daughter cell in each division (Zsok et al. 2024; Khmelinskii et al. 2010) – will lead to significant decreases in nanobody labelling over time. As the reviewer is likely aware, baker’s yeast NPCs – in contrast to mammalian NPCs - remain largely intact during cell division as there is no nuclear envelope breakdown.

**Reviewer #3 (Recommendations for the authors):**

(1) As mentioned above, to assess the general relevance of this tool, it would be informative to verify whether the VHH[Nup84] nanobody can access and detect NPC species under conditions that challenge their structural organization or biogenesis, for example, in nucleoporin mutants or under stress. The authors could, for instance, analyze the localization of VHH[Nup84] in yeast strains harboring clustered NPCs (nup133Δ), or following stresses known to impact NPC organization (e.g., osmotic stress or energy depletion; PMID: 34762489).

We agree with the reviewer that it would be informative to see if VHH[Nup84] can bind its epitope in the context of an altered NPC structure and tried to include such data. Unfortunately, this was not successful, and further efforts are beyond the scope of his study. Following the reviewer’s suggestion, we expressed VHH[Nup84] in *nup*133∆N (nup133∆2-300) (Doye, Wepf, and Hurt 1994) following the experimental set-up in panel A and examined its localization. However, at t=2hrs hardly any nanobody signal was detectable in *nup133∆N* and only after overnight expression nanobody-labelled NPC clusters are detectable (bottom panel A). Considering that expression levels of free mNG are also lower at t=2hrs in *nup133∆N* cells compared to WT cells , it appears that protein expression under the Gal system is generally reduced in a *nup133∆N* background. These expression level differences between *nup133∆N* and WT preclude statements about the accessibility of the Nup84 epitope in *nup133∆N*. We note that nup133∆N cells do not have general mRNA export defects (Doye, Wepf, and Hurt 1994), so other inducible systems may be better suited for such analysis.

(2) Since outer rings are found on both sides of NPCs (i.e., the cytoplasmic and nuclear faces), could the authors indicate whether the VHH[Nup84] nanobody can enter the nucleus and probe the nuclear outer rings? Along these lines, it would be useful to provide a summary of the structural organization of NPCs in the introduction.

Thank you, we have added a sentence on the localization of Nup84 in NPCs in the introduction. Based on what is known about influx (nuclear transport receptor-independent nuclear entry) of proteins with similar size and surface properties (Popken et al. 2015; Timney et al. 2016), the nanobody can rapidly enter the nucleus and hence bind Nup84 on both the nuclear and cytoplasmic side. We have no data to answer if binding might initially be biased towards cytosolic VHH[Nup84] binding the cytoplasmic outer rings.

(3) The authors state that VHH[Nup84] and direct Nup84 detection are indistinguishable (p. 2). Could they provide images of the endogenously tagged Nup84-GFP strain for comparison?

We have now included a pairwise comparison in a Figure 1 – supplement 1.

Minor corrections:

(1) There are a few typos that need correcting: 'Nup84Δ' (p. 1; should read 'nup84Δ') and 'promotor' (p. 2; should read 'promoter').

Thank you, these are corrected

(2) The reference 'Veldsink et al. 2025' (quoted in the PunctaFinder analysis description on page 8) does not appear in the References section.

Thank you, these are corrected.

We thank the reviewer again for the constructive feedback and thoughts.

References

Doye, V., R. Wepf, and E. C. Hurt. 1994. 'A novel nuclear pore protein Nup133p with distinct roles in poly(A)+ RNA transport and nuclear pore distribution', EMBO J, 13: 6062-75.

Khmelinskii, Anton, Philipp J. Keller, Holger Lorenz, Elmar Schiebel, and Michael Knop. 2010. 'Segregation of yeast nuclear pores', Nature, 466: E1-E1.

Popken, Petra, Ali Ghavami, Patrick R. Onck, Bert Poolman, and Liesbeth M. Veenhoff. 2015. 'Size-dependent leak of soluble and membrane proteins through the yeast nuclear pore complex', Molecular Biology of the Cell, 26: 1386-94.

Timney, Benjamin L., Barak Raveh, Roxana Mironska, Jill M. Trivedi, Seung Joong Kim, Daniel Russel, Susan R. Wente, Andrej Sali, and Michael P. Rout. 2016. 'Simple rules for passive diffusion through the nuclear pore complex', Journal of Cell Biology, 215: 57-76.

Zsok, J., F. Simon, G. Bayrak, L. Isaki, N. Kerff, Y. Kicheva, A. Wolstenholme, L. E. Weiss, and E. Dultz. 2024. 'Nuclear basket proteins regulate the distribution and mobility of nuclear pore complexes in budding yeast', Mol Biol Cell, 35: ar143.
